# Prevalence, causes and management outcome of intestinal obstruction in Adama Hospital, Ethiopia

**DOI:** 10.1186/s12893-016-0150-5

**Published:** 2016-06-04

**Authors:** Urgessa Soressa, Abebe Mamo, Desta Hiko, Netsanet Fentahun

**Affiliations:** Department of obstetrics/gynecology and surgery coordinating office, Jimma University specialized hospital, Jimma, Ethiopia; Department of Health Education and Behavioral Sciences, Jimma University, Jimma, Ethiopia; Department of Epidemiology, Jimma University, Jimma, Ethiopia; Department of Health Education and Behavioral Sciences, College of Health Sciences, Jimma University, PO Box 378, Jimma, Ethiopia

**Keywords:** Intestinal obstruction, Prevalence, Causes, Management, Hospital, Ethiopia

## Abstract

**Background:**

In Africa, acute intestinal obstruction accounts for a great proportion of morbidity and mortality. Ethiopia is one of the countries where intestinal obstruction is a major cause of morbidity and mortality. This study aims to determine prevalence, causes and management outcome of intestinal obstruction in Adama Hospital in Oromia region, Ethiopia.

**Method:**

A hospital based cross-sectional study design was used. Data covering the past three years were collected from hospital medical records of sampled patients. The collected data were checked for any inconsistency, coded and entered into SPSS version 16.0 for data processing and analysis. Descriptive and logistic regression analyses were used. Statistical significance was based on confidence interval (CI) of 95 % at a *p*-value of < 0.05.

**Result:**

262 patients were admitted with intestinal obstruction. The prevalence of intestinal obstruction was 21.8 % and 4.8 % among patients admitted for acute abdomen surgery and total surgical admissions, respectively. The mortality rate was 2.5 % (6 of 262). The most common cause of small bowel obstruction was intussusceptions in 48 patients (30.9 %), followed by small bowel volvulus in 47 patients (30.3 %). Large bowel obstruction was caused by sigmoid volvulus in 60 patients (69.0 %) followed by colonic tumor in 12 patients (13.8 %). After controlling for possible confounding factors, the major predictors of management outcome of intestinal obstruction were: duration of illness before surgical intervention (adjusted odds ratio (AOR) = 0.49, 95 % CI: 0.25–0.97); intra-operative findings [Viable small bowel volvulus (SBV) (AOR = 0.08, 95 % CI: 0.01–0.95) and viable (AOR = 0.17, 95 % CI: 0.03–0.88)]; completion of intra-operative procedures (bowel resection & anastomosis (AOR = 3.05, 95 % CI: 1.04–8.94); and length of hospital stay (AOR = 0.05, 95 % CI: 0.01–0.16).

**Conclusion:**

Small bowel obstruction was more prevalent than large bowel obstruction. Intussusceptions and sigmoid volvulus were the leading causes of small and large bowel obstruction. Laparotomy was the most common methods of intestinal obstruction management. Bowel resection and anastomosis was the commonest intra-operative procedure done and is associated with postoperative complications. Wound infection in the affected area should be improved because it is the most common postoperative complication. This can be decreased by appropriate surgical technique and wound care with sterile techniques.

## Background

Intestinal obstruction (IO) is defined as obstruction of the passage of the intestine for its contents [1]. It is potentially risky surgical emergency associated with high morbidity and mortality [2]. Emergency operation being defined as those types of surgeries that should be performed by necessity within 24 h of a patient’s admission, or within 24 h of the development of a specific complication [3]. The research community in both developed and developing countries has investigated this condition [1, 4].

Of all IO, mechanical IO forms an important part of pathologies that necessitate emergency surgical interventions in parts of Asia, including India, Iran and Pakistan [3, 5]. With certain exceptions, mechanical IO can generally be relieved through conservative treatments like naso-gastric tube insertion, intravenous antibiotics or intravenous fluid resuscitation; unrelieved IO necessitates further exploration. Previous studies revealed that repeat IO will recur in about 12 % of patients after primary conservative treatment, and in between 8 and 32 % of patients after operative management for adhesive bowel obstruction [2, 5].

In 80 % of cases, IO occurs in the small bowel, while in 20 % of cases it occurs in the large intestine [1]. There are four cardinal features of IO: colicky abdominal pain, distension, vomiting, and constipation. The presentation of these symptoms is affected by the site and type of obstruction [2, 6].

In rural Africa, acute intestinal obstruction accounts for a great proportion of morbidity and mortality [5], and Ethiopia is one of the countries where intestinal obstruction constitutes a major cause of morbidity and mortality [7]. While some studies have been done to assess prevalence and causes of IO in developed countries, the condition remains largely unstudied in the Ethiopia context. With only a few studies conducted in north and central Ethiopia [8, 9], there is a lack of research about the prevalence and causes of IO in Ethiopia, particularly in the western and eastern parts of the country. Furthermore, there is no recently published literature that has explored IO in rural and regional hospitals. Thus, this study was conducted to fill this information gap and generate base line information about prevalence, causes and management outcome of IO in rural Ethiopia.

The results of this study will provide epidemiological and clinical information that will serve as an essential input for policy makers to design proper strategies to address IO. Moreover, the study results will serve as references for those who want to undertake research on the prevalence and causes of IO.

## Methods

### Study design and period

Hospital based cross sectional study design was conducted at Adama Hospital Medical College, drawing upon medical records dated January 1, 2011 to December 30, 2013. The data collection period was from February 1–15, 2014.

### Study population

The study population included all patients admitted with diagnosis of IO at Adama Hospital Medical College during the three year period. The following types of patients were included: patients whose IO was managed conservatively without operation; patients who underwent an operation for IO; and patients who died due to a confirmed diagnosis of IO. Patients who had incomplete records (i.e., important information on causes and management outcome variables), or whose records were lost were excluded from further analysis.

### Participant selection

A subsample of data was obtained from all patients admitted to the surgical ward of Adama Hospital Medical College with an IO diagnosis and treated from January 1, 2010 to December 30, 2012. Therefore, 262 patients were admitted for IO during the study period, the data of 242 patients were included for further study.

### Data collection methods

The data were collected by reviewing the registration books using structured checklists. A check list was developed in the English language to collect important information such as age, sex, admission diagnosis, intra-operative findings, intra-operative procedures, duration of presentation, causes of IO, postoperative complications and management outcome. For data collection, two clinical nurses were recruited who were not part of the Adama Hospital Medical College staff. The Principal Investigator provided training for data collectors on how to complete the checklist, as well as the importance of data quality and relevance of the study. Another clinical nurse (a first-degree holder) supervised the data collection activities, ensuring the consistency and completeness of the checklist and providing appropriate support. The Principal Investigator oversaw the daily activities of data collectors and supervisor.

### Data collection techniques and measurements

Patients that were admitted to surgical wards with the diagnosis of IO were initially identified from admission logbooks of surgical wards and operation theaters of the hospital from which the card number of patients was obtained. Using the cards as a reference, patients were identified, and relevant information was collected about patients admitted with the diagnosis of IO.

The concepts measured in this study were defined based on a review of relevant literature that has assessed similar key variables as well as earlier studies on managing outcome of IO in health institutions. For the purposes of this study, conservative management includes the management of patients with partial bowel obstruction or recurrent adhesive obstruction; it also includes the management of patients during the early postoperative period with naso-gastric tube NGT suction, intravenous (IV) fluids and frequent clinical reassessment. (The purpose of frequent clinical assessment is to rule out bowel strangulation, which may need operative management.) Operative management includes surgical exploration or operations performed on the abdomen to relieve the causes of obstruction.

A favorable outcome was achieved if patients did not develop either postoperative complication or death after conservative or operative management of IO. If the patient developed one or more postoperative complications (including wound infection, facial dehiscence, anastomotic leakage, developed septic shock, pelvic collection and pneumonia) and/or death this was considered an unfavorable outcome of IO.

### Data processing, analysis, interpretation and presentation

Data were cleaned, coded and entered into the computer using Epi Data version 3.1, and were subsequently exported to SPSS version 16.0 for further analysis. Results were presented using frequency tables, graphs and percentage. Bivariate logistic regression analysis was done to determine crude statistical associations between independent variables and dependent variables (management outcome of IO). Variables with a *p*-value of less than 0.025 in binary logistic regression analysis were considered as a candidate to be entered into multivariate logistic regression. Multivariable analyses were used to isolate the independent predictor of management outcome of IO. Statistical significance was based on a *p*-value of < 0.05 with a confidence interval (CI) of 95 %.

### Data quality management and collection

Before data collection, the checklists were assessed by principal investigator as well as facilitators and supervisors were trained for two days. To avoid interpersonal variation between data collectors, the same two data collectors were retained throughout the data collection process. Regular daily supervision by the Principal Investigator supported the consistency and completeness of checklists. The completed checklists were checked for their completeness and consistency at every step of data collection. After data collection and before starting data analysis, checklist completeness was rechecked.

## Results

### Demographic characteristics

Over the study period, 5500 patients were admitted to the surgical ward, of which 1200 patients were admitted with diagnoses pertaining to acute abdomen conditions. Two hundred sixty two patients were admitted to the surgical ward with IO of whom data of 242 (92.4 %) patients were retrieved for further analysis. The minimum age was 1 month and maximum was 85 years with a mean of 32.8 years (standard deviation (SD) ± 22.6) years. The study revealed that 191 (78.9 %), 157 (64.9 %) and 90 (36.8 %) of the study participants were males, from rural areas, and farmers, respectively.

### Types and prevalence of intestinal obstruction

The prevalence of IO was 21.8 % among patients admitted with the acute abdomen conditions, and 4.8 % among total surgical admission patients. Out of the 242 patients with IO who underwent further analysis, 64 % had cases of small bowel obstruction and 36.0 % had cases of large bowel obstruction. Of these 242 patients, 13.6 % had a previous history of abdominal operation: in 30 patients (12.4 %) the operation was for adhesion, and in ## patients (31.2 %) the operation was for acute appendicitis (Table 1).

**Table 1:** Types of intestinal obstruction and its prevalence in Adama hospital medical college, February, 2014

Variable	Frequency	Percent
Kinds of IO depending on etiology		
Mechanical IO	236	97.5
Paralytic ileus	6	2.5
Total	242	100.0
Types of IO depending on bowel involvement		
SBO	155	64.0
LBO	87	36.0
Total	242	100.0
Previous history of abdominal operation		
No	211	87.1
Yes	33	13.6
Total	242	100.0
Previous history of Adhesion		
No	212	87.6
Yes	30	12.4
Total	242	100.0
Previous history of appendectomy		
No	239	98.8
Yes	3	1.2
Total	242	100.0

### Etiology, intra-operative findings and procedures completed

In this study, most small bowel obstruction was found to be secondary to intussusceptions (in 30.9 % of the cases) or volvulus (in 30.3 % of the cases). Large bowel obstruction was mainly caused by sigmoid volvulus (69.0 %) and colonic tumor (5.3 %). As expected, the main intra-operative finding was intussusceptions, which accounted for 21 % followed by adhesion and bands in 18.4 % (Table 3). The most common intra-operative procedure was resection and anastomosis, which accounted for 40.5 %, followed by manual reduction and adhesion release, each accounting for 17.4 % (Table 2).

**Table 2:** Causes of intestinal obstruction and intra-operative finding in Adam hospital medical college, February, 2014

Variables	Frequency	Percent
Causes of Small bowel obstruction		
Intussusceptions	48	30.9
Small bowel volvulus	47	30.3
Adhesion	42	27.1
Hernia	9	5.8
Iliosigmoidal knotting	5	4.3
Appendicitis	2	1.3
Intestinal TB	1	0.6
Mickel’s diverticulitis	1	0.6
Total	155	100.0
Causes of large bowel obstruction		
Sigmoid volvulus	60	69.0
Colonic tumor	12	13.8
Intussusceptions	8	9.2
Iliosigmoidal knotting	5	5.7
Fecal impaction	2	2.3
Total	87	100.0
Intra-operative finding		
Intussusceptions	48	21.0
Viable sigmoid volvulus	42	18.4
Adhesion & bands	42	18.4
Viable small bowel volvulus	31	13.6
Gangrenous SBV	18	7.9
Gangrenous sigmoid volvulus	18	7.9
Colonic cancer	12	5.3
Iliosigmoidal knotting	5	2.2
Intestinal TB	4	1.7
Meckel’s diverticulitis	3	1.3
Others	5	2.2
Total	228	100.0

### Management outcome of intestinal obstruction

**Fig. 1:**
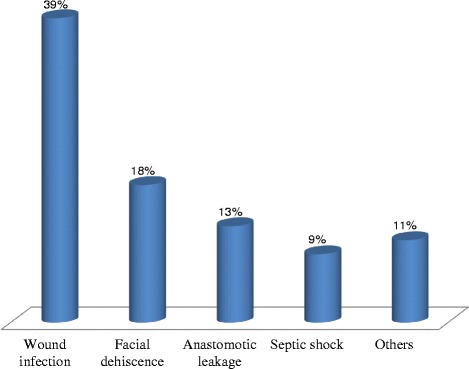
Prevalence of postoperative complication of intestinal obstruction patients in Adama hospital medical college February, 2014

Nearly 94.2 % of IO cases were managed by surgical procedure, whereas simple conservative management alone (i.e., naso-gastric tube insertion, intravenous antibiotics and intravenous fluid resuscitation) were applied in 5.8 % of cases. Males in 65.8 % of the cases and females in 20.2 % were managed by operation. Of the patients that underwent laparatomy, 56 patients (24.6 %) developed an unfavorable outcome. Among these, 22 patients (39.3 %) developed wound infection, 10 (17.8 %) had facial dehiscence, 7 (12.5 %) had anastomotic leakage,5 (8.9 %) developed septic shock and 6 (10.7 %) developed other complications like pelvic collection or pneumonia (Fig. 1).

The minimum duration of illness before arrival was 2 h and the maximum was 78 h (mean 28.63 h; SD ± 17.66). One hundred nineteen patients (49.2 %) presented within 24 h, whereas 50.8 % presented after 48 h. The minimum duration of hospital stay was 2 days and the maximum duration was 30 days with an average of 9.54 days (SD ± 4.51) . About 120 patients (49.6 %) stayed in hospital for between 1 and 7 days, whereas 122 patients (50.4 %) stayed more than 7 days. Out of the 242 patients with IO, 234 (96.7 %) improved and were discharged; 6 (2.5 %) died.

### Predictors of management outcome of intestinal obstruction

Variables with a *p*-value of less than 0.025 in binary logistic regression analysis were entered in multivariate logistic regression to isolate the independent predictor of management outcome of IO. This allowed us to control for all variables that may be potential confounding factors. The result showed that duration of illness before surgical intervention had a significant statistical association with the management outcome of patients. Patients who presented within 24 h of symptom presentation are less likely to develop an unfavorable outcome, compared with patients who presented after 24 h (adjusted odds ratio (AOR) = 0.49, 95 % CI: 0.25–0.97, *p*-value = 0.01).

Patients with viable small bowel volvulus and viable sigmoid volvulus were less likely to develop an unfavorable outcome compared with patients with gangrenous small bowel volvulus ((AOR = 0.08, 95 % CI: 0.01–0.95, *p*-value = 0.04) and (AOR = 0.17, 95 % CI: 0.03–0.88, *p*-value = 0.03), respectively).

Bowel resection and anastomosis showed a three folds risk of developing unfavorable outcome compared with patients without resection and anastomosis of bowel (AOR = 3.05, 95 % CI: 1.04–8.94, *p*-value = 0.02). Adhesion release also had a significant statistical association with management outcome (AOR = 0.09, 95 % CI: 0.01–0.69, *p*-value = 0.02). Intra-operative manual reduction and length of hospital stay had a significant statistical association with management outcome (AOR = 0.15, 95 % CI: 0.03–0.68, *p*-value = 0.01) and (AOR = 0.05, 95 % CI: 0.01–0.16, *p*-value = 0.001) respectively. Patients who had stayed for less than 7 days were less likely to develop an unfavorable outcome compared with patients who stayed for more than 7 days (AOR = 0.05, 95 % CI: 0.01–0.16, *p*-value = 0.001) (Table 3).

**Table 3:** Factors associated with management outcome of intestinal obstruction in Adama hospital medical college, February, 2014

Variables	Management outcomes		COR (95 % CI)	AOR (95 % CI)	*P*-value
	Favorable [N]	Unfavorable [N]			
Age					
<14 years	52	8	0.287(0.11–0.76)	0.99(0.11–2.13)	0.998
14–24 years	21	7	0.62(0.21–1.79)	0.59(0.109–3.21)	0.543
25–34 years	32	12	0.70(0.28–1.74)	0.86(0.23–3.17)	0.828
35–44 years	27	5	0.34(0.11–1.08)	0.35(0.08–1.48)	0.158
45–55 years	45	9	0.64(0.242–1.73)	0.28(0.07–1.08)	0.066
>55 years	28	15	1	1	
Occupation					
Farmer	64	26	1	1	
Merchant	18	7	0.95(0.35–2.56)	1.93(0.56–6.56)	0.291
House wife	14	13	2.28(0.94–5.52)	3.28(0.97–11.11)	0.056
Student	18	1	0.14(0.02–1.07)	0.97(1.87–2.61)	0.998
Governmental employer	23	2	0.21(0.05–0.97)	0.14(0.02–1.09)	0.061
Duration of illness					
≤24 h	101	18	0.39(0.21–0.74)	0.49(0.25–0.97)	0.014*
>24h	85	38	1	1	
Intra-operative finding					
Gangrenous SBV	9	9	1	1	
Gangrenous SV	8	10	1.25(0.33–4.63)	1.95(0.34–10.95)	0.447
Adhesion & bands	28	14	0.50(0.16–1.54)	5.90(0.72–4.80)	0.098
Viable SBV	29	2	0.07(0.01–0.37)	0.08(0.01–0.95)	0.046*
Viable SV	34	7	0.21(0.06–0.71)	0.17(0.03–0.88)	0.035*
Intussusceptions	49	7	0.14(0.04–0.48)	0.49(0.07–3.22)	0.460
Colonic Cancer	8	4	0.50(0.11–2.27)	0.27(0.04–1.64)	0.157
Hernia	8	1	0.12(0.01–1.216)	0.12(0.01–1.65)	0.114
Illiosigmoidal knot	4	1	0.25(0.023–2.69)	1.40(0.04–43.13)	0.844
Intra-operative procedure done					
Resection& Anastomosis					
Yes	63	35	3.02(1.61–5.69)	3.05(1.04–8.94)	0.041*
No	109	21	1	1	
Adhesion release					
Yes	33	9	0.83(0.37–1.86)	0.09(0.01–0.69)	0.020*
No	139	47	1	1	
Manual Reduction					
Yes	41	2	0.12(0.02–0.52)	0.15(0.03–0.68)	0.014*
No	131	54	1	1	
Hospital stay					
≤ 7 days	117	3	0.03(0.01–0.11)	0.05(0.01–0.16)	0.001*
>7 days	69	53	1	1	

*Significant at *p*-value<0.05 1 is reference

*SBV* small bowel volvulus, *SV* sigmoid volvulus, *SBO* small bowel obstruction, *IO* intestinal obstruction

## Discussion

This study revealed the prevalence of IO to be 4.8 % among all surgical patients admitted to surgical ward and 21.8 % among patients admitted with a diagnosis of acute abdomen with subsequent surgical management. Indeed, world wide it has been estimated that 1 % of all hospitalizations, 3 % of emergency surgical admissions to general hospitals and 4 % of major colostomies are secondary to IO. This study confirmed previous findings that between 12 and 17 % of patients are admitted for small bowel obstruction within two years of their index operation, while approximately 3 % require an operation to treat an established small bowel obstruction [10]. Currently, many patients that present to general surgery services with acute abdomen conditions are thought to have IO [8]. While IO is rare in the USA and Western Europe, it is common in Pakistan and other tropical countries. It is the leading cause of acute abdomen complaints in several African countries, including Ethiopia [1, 6, 7]. In general, there are wide variations in the prevalence of IO throughout the world depending on ethnicity, age group, dietary habits, and geographic location, among other factors. It varies from country to country and area to area in the same country [11].

Intra-operative finding like, viable small bowel volvulus (SBV) and viable sigmoid volvulus were found to be statistically significant with management outcomes. Patients with viable SBV or viable sigmoid volvulus are less likely to develop an unfavorable outcome as compared with patients with gangrenous small bowel volvulus which is similar with other studies [12, 13].

Similar studies also reported that small bowel obstruction was the most common type of IO whereas large bowel obstruction was relatively less common. Some other studies revealed that small bowel obstruction has taken the lion share and various studies agreed for the fact that small bowel obstruction is more prevalent than large bowel obstruction [8, 14]. This fact may help us to confirm that socio-economic factors and diet might be responsible for the causes of this problem in some developing countries. A previous study in an Ethiopian hospital found that small bowel and large bowel obstruction account for 52.3 and 46.7 % respectively [7]. Mechanical IO were most common obstruction in our study, whereas adynamic obstruction was less prevalent. A study in Pakistan also confirmed that paralytic ileus was common and causes of small bowel obstruction were mostly due to intussusceptions and volvulus [1].

Intra-operative procedures like bowel resection and anastomosis have significant statistical association with management outcome. Bowel resection and anastomosis has a three times high risk of developing an unfavorable outcome compared with patients without resection and anastomosis of bowel.

Adhesion release and manual reduction intra-operatively also demonstrated a significant statistical association with management outcome. According to this study, the common causes of intestinal obstruction were mostly due to adhesion whereas obstructed/strangulated hernia was less prevalent. A study conducted in Western Sudan, showed obstructed/strangulated hernia to be more prevalent than adhesion, while SBV was found to be the least prevalent by far [15]. Similarly, a study conducted in Uganda also confirmed that the four leading causes of obstruction were hernias, adhesions, volvulus and intussusceptions [16]. A six years study of 56 cases of IO seen and treated at Asir Central Hospital in Saudi Arabia found that adhesion was the most common cause of IO, however, hernias were a leading cause of obstruction in most African centers. This might imply that the culture, food, age of the patients and geography have huge influences on the prevalence of obstruction. A study also confirmed that IO is almost certainly affected by the paucity of surgical services in the region [17]. Intussusception was the most common IO in our study, especially in children, indicates a possible association with upper respiratory tract infections. Patients’ large bowels were obstructed secondary to sigmoid volvulus in 69.0 % of the cases. This was not surprising, as sigmoid volvulus has been reported to be a frequent cause of IO in a African countries [17]. A study reported that sigmoid volvulus was the leading cause of IO in the northern part of Ethiopia [7]. Sigmoid volvulus is also the leading cause of large bowel obstruction in most of the sub-Saharan region [9, 18].

Duration of illness before surgical intervention has significant statistical association with management outcome of patients. Patients who presented within 24 h duration of illness are less likely to develop unfavorable outcome compared with patients who presented after 24 h. A study in Rwanda indicated that more than three-fourths of patients presented after 24 h [19], however, in our study half of the patients presented within 24 h. Delayed presentation and/or surgical intervention frequently results in relatively poor surgical outcomes and/or longer hospital stays [17].

Our study also revealed that patients who stayed for less than 7 days were less likely to develop unfavorable outcomes compared with patients who stayed for more than 7 days. By comparison, one study of pattern of acute abdomen in Butajira, Ethiopia showed that the average hospital stay was 9 days and a third of patients developed one or more acute complications [9, 20]. The difference in IO outcome may be associated with late duration of patients’ illness to hospital due to lack of awareness about the burden and impacts of the problem. The potential reasons for lower mortality rate in our study may be due to early intervention of the obstruction before complications occur and adequate preoperative resuscitation which might be expected to decrease mortality.

As to the management, laparotomy or surgical incision was the most common method of IO management in this investigation; our results indicated slightly higher rates than previous research conducted in Nigeria and USA [21, 22]. The outcome of laparotomy might be affected by different factors, such as cause of obstruction, duration of illness, age, presence of peritonitis and complication detection time.

This study was subject to certain potential limitations. The use of secondary data confers lack of oversight about how the original data were collected. For instance, some patient information may have been missed or incorrectly recorded. This in turn may lead to under or over estimations of the finding. As much as possible, we tried to manage and minimize these limitations through careful selection of records of the patients.

## Conclusions

In conclusion, small bowel obstruction was more prevalent than large bowel obstruction in this study. Intussusceptions and sigmoid volvulus were the leading causes of small and large bowel obstruction, respectively. Laparotomy was the most common means of IO management, while bowel resection and anastomosis was the most common intra operative procedure. The most commonly encountered postoperative complications were wound infection followed by facial dehiscence.

Our findings suggest that health professionals in the hospital and district should increase public awareness on IO by providing appropriate health information. Physicians should diagnose intestinal early and appropriate interventions should be taken on time before the intestine develops gangrene. Wound infection in the study area should be improved because it is the most common postoperative complication in the area. This can be decreased by appropriate surgical technique and wound care with sterile techniques. Card room staff should improve record keeping in the hospital because some handwriting was not readable and some charts were lost. Further research using prospective study design is warranted as a way to overcome the limitations of secondary data in the current retrospective research that preclude generalization to the whole population.

## Abbreviations

AOR, adjusted odds ratio; CI, confidence interval; COR, crude odds ratio; IO, intestinal obstruction; LBO, large bowel obstruction; MPH, Master of Public Health; SBO, small bowel obstruction; SBV, small bowel volvulus; SD, standard deviation; SPSS, Statistical Packages for Social Science; WHO, World Health Organization
